# HOMA-IR, an independent predictor of advanced liver fibrosis in metabolic-dysfunction associated fatty liver disease: a cross-sectional study in Egyptian patients

**DOI:** 10.1038/s41598-025-15425-7

**Published:** 2025-08-24

**Authors:** Yasser Fouad, Ziyan Pan, Shaymaa Nafady, Alaa M. Mostafa, Asmaa Bakr, Mahmoud Hagag, Ahmed Gomaa, Samy Zaky, Mohammed Eslam

**Affiliations:** 1 Department of Gastroenterology, Hepatology and Endemic Medicine, Faculty of Medicine, Minia University, Main Road, Cairo, Minia, 11432 Egypt; 2 Storr Liver Centre, Westmead Institute for Medical Research, Westmead Hospital and University of Sydney, Sydney, NSW Australia; 3 Department of Gastroenterology, Hepatology and Infectious Diseases, Beni-Suef University, Beni-Suef, Egypt; 4 Departmeent of Gastroenterology, Hepatology and Infectious Diseases, Al-Azhar University, Cairo, Egypt; 5 Department of Gastroenterology, Hepatology, Ministry of Health, Cairo, Egypt; 6 Department of Tropical Medicine, Fayoum University, Fayoum, Egypt

**Keywords:** MAFLD, Fatty, HOMA-IR, Insulin resistance, Gastroenterology, Medical research, Risk factors

## Abstract

While metabolic dysfunction-associated fatty liver disease (MAFLD) includes the homeostatic model assessment for insulin resistance (HOMA-IR) as one of the criteria to define metabolic dysregulation, the newly proposed metabolic dysfunction-associated steatotic liver disease (MASLD) has removed this criterion. We investigated whether the HOMA-IR can serve as an independent predictive marker for significant fibrosis in subjects with MAFLD. This is a cross-sectional multicenter study. We enrolled a total of 364 patients diagnosed with MAFLD. We conducted a multiple logistic regression analysis to assess the relationship between HOMA-IR and advanced stages of liver fibrosis (F ≥ 2), as assessed by the FIB-4 score and liver stiffness measurement (LSM). Each unit increase in insulin resistance, as measured by HOMA-IR, was associated with a 16% higher likelihood of displaying significant fibrosis, as determined by a non-invasive scoring test, regardless of diabetes or BMI status. HOMA-IR was independently associated with significant fibrosis in non-diabetic (OR: 1.14, 95% CI: 1.07–1.21, P < 0.001) and diabetic (OR: 1.03, 95% CI: 1.00–1.06, P = 0.03) patients. Moreover, significant fibrosis in lean was independently linked to HOMA-IR (OR: 1.06, 95% CI: 1.01–1.12, P = 0.03) and non-lean (OR: 1.04, 95% CI: 1.02–1.07, P < 0.001) patients. Insulin resistance measured by HOMA-IR should be assessed in patients with MAFLD as a key factor of disease progression and incorporated into the disease diagnostic criteria.

## Introduction

Metabolic dysfunction-associated fatty liver disease (MAFLD) is a common liver disease that affects more than 30% of adults worldwide. Its occurrence is increasing due to the growing rates of obesity and diabetes^1–3^. MAFLD is associated with elevated mortality rates attributed to both cardiovascular disease and liver disease^4,5^. Evaluating and early detection of the development of fibrosis are crucial in predicting the prognosis of patients with MAFLD^6,7^.

In 2020, there was a significant change in the way fatty liver disease due to metabolic dysfunction is diagnosed. The definition has been revised to shift from diagnosis of exclusion of non-alcoholic fatty liver disease (NAFLD) to positive diagnosis, using a set of practical and affirmative diagnostic criteria. The diagnostic criteria of MAFLD^8–10^ have been robustly shown to be highly effective in identifying both the liver-related and non-liver-related effects of the disease, even in individuals with other liver diseases^11^. These criteria are based on the presence of hepatic steatosis in conjunction with overweight status, Type 2 Diabetes Mellitus (T2DM), or at least two metabolic risk factors, such as elevated HOMA-IR or high-sensitivity C-reactive protein (hs-CRP).

In 2023, a Delphi consensus statement by a panel of international experts proposed another name, metabolic dysfunction-associated steatotic liver disease (MASLD)^12^, along with modified corresponding diagnostic criteria. The diagnostic criteria for MASLD necessitate the presence of at least one metabolic risk factor to identify metabolic dysregulation, even in lean patients. In addition, the homeostasis model assessment of insulin resistance (HOMA-IR) and C-reactive protein, which were included in the initial criteria for MAFLD, were excluded based on these criteria.

Insulin resistance (IR) is a condition that affects the body’s ability to use insulin effectively. This is a key factor in the development and progression of metabolic dysfunction and MAFLD. The homeostasis model assessment of insulin resistance (HOMA-IR) is a useful and direct method for evaluating insulin resistance and beta cell function in both diabetic and non-diabetic individuals^13^. HOMA-IR is calculated using fasting glucose and insulin levels, and it provides an estimate of insulin resistance and can identify individuals who are at risk of developing diabetes and other metabolic disorders^13^.

Significant fibrosis is the strongest predictor of increased risk of liver-related complications^14^. Given the close association between insulin resistance and MAFLD, it is crucial to assess the predictive value of HOMA-IR and its correlation with significant fibrosis in patients with MAFLD. This knowledge can inform the decision on the appropriateness of various definitions of fatty liver disease associated with metabolic dysfunction.

This study aimed to assess the influence of insulin resistance on severe liver fibrosis (F ≥ 2), using transient elastography. Additionally, the study aimed to analyze the specific nature of this correlation within different subgroups.

## Methods

### Cohort

A multicenter cross-sectional study evaluating 364 consecutive Middle Eastern Egyptian patients with MAFLD, diagnosed according to the established international criteria, in whom HOMA-IR values have been measured and liver stiffness has been assessed in this multicentre study. Diagnosis of MAFLD population based on imaging (VCTE) with metabolic criteria: overweight or obesity, T2DM, or lean/normal weight with at least two metabolic risk abnormalities; (Waist circumference ≥ 102/88 cm in Caucasian men and women), (blood pressure ≥ 130/85 or told by the doctor to have hypertension), (Plasma Triglyceride level is ≥ 1.7 mmol/dL) or specific drug treatment, (Plasma HDL-Cholesterol level < 1.0/1.3 mmol/dL for men and women). HOMA-IR ≥ 2.5, and plasma Hs-CRP ≥ 2 mg/L ^8^. Patients with secondary causes of steatosis, drug-induced liver disease, alcoholic liver disease, or viral hepatitis were excluded.

Written informed consent was obtained from each patient. This study was conducted in concordance with the ethical guidelines of the Declaration of Helsinki and with the approval of the ethics and research committees of each centre (approval no 1546).

### Anthropometric evaluations

Before conducting liver stiffness testing, a thorough physical examination was performed for each patient. The BMI, defined as ≥ 25 kg/m2, was calculated by dividing the weight (in kilograms) by the square of the height (in meters). However, patients were categorized as lean if BMI < 25 kg/m2 in concordance with standard definition. Waist circumference was recorded using standard protocol at the midpoint between the lower margin of the last rib and the top iliac crest. The history of diagnosis of arterial hypertension and diabetes was established based on internationally recognized criteria.

#### Laboratory assessment

During the morning, venous blood samples were collected following a 12-h overnight period of fasting. The laboratory assessment for all patients consisted of a comprehensive analysis of blood cell count, hemoglobin level, and platelet count, as well as measurements of aspartate aminotransferase (AST), alanine aminotransferase (ALT), albumin, fasting blood glucose, serum cholesterol, high-density lipoprotein cholesterol, triglyceride level, and fasting immunoreactive insulin (IRI) levels.

The FIB-4 index was calculated using the formula: age (in years) multiplied by AST (in U/L), divided by the product of platelet count (in 10^9/L), and the square root of ALT (in U/L). The measurement of serum insulin was conducted using standard laboratory procedures. The calculation of insulin resistance was determined using the HOMA-IR method, which involves multiplying the fasting insulin level (measured in mU/mL) by the fasting plasma glucose level (measured in mmol/L) and then dividing the result by 22.5.

### Liver fibrosis assessment

Trained technicians conducted vibration-controlled transient elastography (VCTE) Fibroscan. The present research only included individuals who had undergone comprehensive examinations with at least 10 stiffness measurements and had an interquartile range/median ratio of liver stiffness less than 30%. liver stiffness of ≥ 8 kPa is used as a cut-off of significant fibrosis^15,16^.

### Statistical analyses

The statistical analyses were performed using the IBM SPSS Statistics software package (SPSS, version 25). The continuous variables are shown as means with their corresponding standard deviations and were assessed by Student’s *t*-test. The qualitative data is displayed as numbers accompanied by their corresponding percentages and evaluated using the chi-square test. Data for certain variables, including waist circumference, cholesterol, and triglyceride, were analyzed using available-case analysis without imputation. A multivariate logistic regression model was utilized to analyze the relationship between advanced fibrosis and confounding factors while accounting for patient characteristics. Variables were selected for the multivariate logistic regression model according to their clinical and statistical significance for the outcome of interest, considering the individual’s diabetes state and BMI. We also performed diagnostic checks for multicollinearity using the Variance Inflation Factor (VIF < 5 for all included variables). Sensitivity analysis (ROC curve) was conducted to evaluate the correlation between insulin resistance and liver fibrosis.

## Results

### Patient characteristics

The study comprises 364 patients with MAFLD. Their clinical characteristics are depicted in Table 1. The mean age of the patients was 48.15 ± 11.88 years 139 (38.2%) of them were men and 118 (32.4%) had diabetes. The mean BMI of the patients was 31.26 ± 5.45, the ALT level was 35.52 ± 20.98, and the fasting HOMA-IR was 10.95 ± 14.87. 170 patients (46.7%) had significant fibrosis based on liver stiffness measurement and 140 (38.5%) based on FIB-4 score.

**Table 1:** Baseline characteristics of the cohort.

Total cohort (n = 364)		
Characteristics	Mean ± SD / number	Range (min–max) / %
Age (year)	48.15 ± 11.88	16–75
Male (%)	139	38.2%
Diabetes (%)	118	32.4%
BMI (kg/m^2^)	31.26 ± 5.45	20.30–50.47
Waist circumference (cm)	104.56 ± 11.04	78–139
ALT (U/L)	35.52 ± 20.98	3.35–163.00
AST (U/L)	47.64 ± 27.76	10.00–150.00
Platelet (1000 cells/uL)	237.55 ± 70.43	3.34–495.00
HOMA-IR	10.95 ± 14.87	0.10–72.60
Hemoglobin A1c	6.40 ± 2.54	1.04–34.90
Total cholesterol (mg/dL)	191.48 ± 39.233	108–299
LDL-cholesterol (mg/dL)	132.87 ± 140.13	42–1415
HDL-cholesterol (mg/dL)	44.63 ± 11.07	25–74
Triglyceride (mg/dL)	148.66 ± 66.16	58–395
Albumin (g/dL)	4.19 ± 0.47	3.10–5.50
Total bilirubin (mg/dL)	0.79 ± 0.49	0.20–2.20
INR	1.01 ± 0.08	0.90–1.20
High-sensitive C-reactive protein (mg/L)	19.82 ± 53.11	0.20–259.00
Lymphocyte (%)	7.06 ± 2.21	2.1–11.4
ALP (IU/L)	77.16 ± 32.99	31.00–149.00
FIB-4	2.93 ± 17.56	0.14–336.00
Significant fibrosis ≥ 8 kPa (LSM)	170	46.7%
Significant fibrosis (FIB4)	140	38.5%

### Insulin resistance is independently associated with significant fibrosis in the overall cohort

**Fig. 1:**
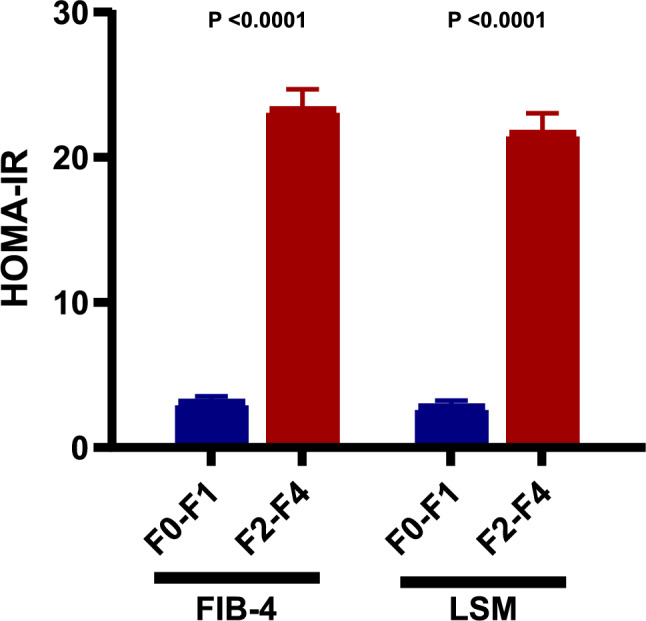
Association between the HOMA-IR and severe fibrosis (F ≥ 2) evaluated by FIB-4 index and liver stiffness measurement (LSM).

**Fig. 2:**
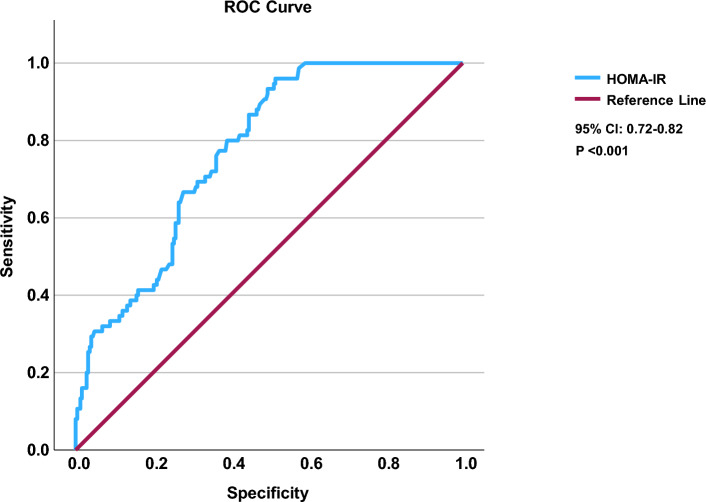
ROC curve for HOMA-IR in the prediction of severe fibrosis (F ≥ 2) measured by liver stiffness measurement (LSM). Area under curve [AUC] is 0.77.

In univariate analysis, older age, presence of type 2 diabetes, elevated ALT, AST, HOMA-IR, Haemoglobin A1c, and lower platelets count were significantly associated with significant fibrosis assessed by kPa (Table 2). Elevated HOMA-IR was associated with a higher risk of significant hepatic fibrosis, as evaluated by the FIB-4 index and liver stiffness (Fig. 1). After adjusting for confounding, a subsequent analysis was conducted using multiple logistic regression analysis. Table 3 shows the results of the independent predictors for diagnosing significant fibrosis based on liver stiffness measurement. It was found that for each unit increase in HOMA-IR, the independent odds of having significant fibrosis increased by 16% (odds ratio [OR], 1.16; 95% confidence interval [CI], 1.10–1.22; p < 0.001). Based on the ROC curve, the optimal cutoff in the HOMA-IR for predicting the presence of severe fibrosis (≥ F2) was 0.77 (95% CI: 0.72–0.82; p < 0.001) (Youden index: 0.379) with 95.2% sensitivity, 42.7% specificity, 52.7% PPV, and 33.3% NPV (Fig. 2).

**Table 2:** Univariate analysis of factors associated with the presence of significant fibrosis measured by FibroScan.

Characteristics	F0-F1	F2-F4	*p*
N	153	170	
Age (year) mean ± SD	43.25 ± 11.08	53.05 ± 10.23	< 0.001
Male N (%)	49 (32.0%)	64 (37.6%)	0.13
Diabetes N (%)	21 (13.7%)	91 (53.5%)	< 0.001
BMI (kg/m^2^) mean ± SD	31.11 ± 5.37	30.39 ± 5.05	0.88
Waist circumference (cm) mean ± SD	103.27 ± 11.28	105.08 ± 10.44	0.21
ALT (U/L) mean ± SD	35.46 ± 24.54	36.50 ± 18.25	0.04
AST (U/L) mean ± SD	32.04 ± 19.82	65.92 ± 24.86	< 0.001
Platelet (1000 cells/uL) mean ± SD	272.38 ± 62.45	198.98 ± 56.51	< 0.001
HOMA-IR mean ± SD	2.81 ± 5.44	20.17 ± 16.94	< 0.001
Hemoglobin A1c (%) mean ± SD	4.96 ± 1.18	7.68 ± 2.86	< 0.001
Total cholesterol (mg/dL) mean ± SD	195.91 ± 40.80	185.56 ± 33.23	0.57
LDL-cholesterol (mg/dL) mean ± SD	162.44 ± 231.72	117.62 ± 26.80	0.63
HDL-cholesterol (mg/dL) mean ± SD	48.25 ± 13.09	39.60 ± 8.83	0.05
Triglyceride (mg/dL) mean ± SD	158.35 ± 76.69	131.36 ± 48.10	0.31
Albumin (g/dL) mean ± SD	4.22 ± 0.44	4.28 ± 0.48	0.32
Total Bilirubin (mg/dL) mean ± SD	0.53 ± 0.25	1.13 ± 0.73	0.39
INR mean ± SD	0.98 ± 0.03	1.02 ± 0.10	0.38
High-sensitive C-reactive protein (mg/L) Mean ± SD	6.56 ± 10.69	14.52 ± 17.12	0.82
Lymphocyte (%) mean ± SD	6.89 ± 1.97	5.69 ± 2.01	0.19
ALP (IU/L) mean ± SD	70.01 ± 31.21	74.73 ± 35.97	0.62

**Table 3:** Multivariate analysis of factors independently associated with the presence of significant fibrosis measured by FibroScan.

Variables	OR (95% CI)	p-value
Age	1.05 (1.02–1.08)	< 0.001
Male	1.28 (0.65–2.52)	0.48
BMI	0.99 (0.93–1.05)	0.79
Platelet	0.99 (0.98–0.99)	< 0.001
ALT	1.00 (0.99–1.02)	0.51
HOMA-IR	1.16 (1.10–1.22)	< 0.001

*OR* odds ratio, *CI* confidence interval.

### Impact of insulin resistance on fibrosis according to the presence of diabetes

**Fig. 3:**
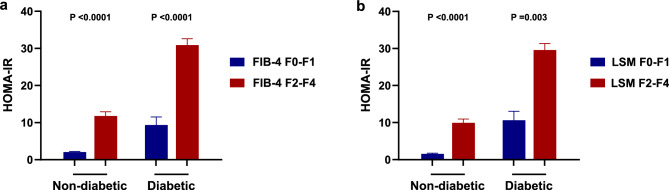
Association between the HOMA-IR and severe fibrosis (F ≥ 2) evaluated by FIB-4 index and liver stiffness measurement (LSM), stratified by the presence of diabetes.

Insulin resistance calculated by the HOMA-IR method correlates closely with the gold standard hyperinsulinemic/euglycemic clamp method in both diabetic and non-diabetic subjects14. Since, the presence of diabetes may be a confounding factor, in a subsequent analysis we explored whether the relationship between fibrosis severity and IR was maintained in non-diabetic patients. As shown in Fig. 3, HOMA-IR was significantly associated with fibrosis severity (kPa and FIB-4) in both diabetic and non-diabetic patients with MAFLD. In multiple logistic regression analysis, HOMA-IR was independently linked to significant fibrosis in non-diabetic (OR: 1.14, 95% CI: 1.07–1.21, p < 0.001) and diabetic (OR: 1.03, 95% CI: 1.00–1.06, p 0.03) patients (Table 4).

**Table 4:** Multivariate analysis of factors independently associated with the presence of significant fibrosis measured by FibroScan stratified by the presence of diabetes.

Non-diabetic			Diabetic		
Variables	OR (95% CI)	p-value	Variables	OR (95% CI)	p-value
Age	1.04 (0.99–1.08)	0.05	Age	1.03 (0.99–1.08)	0.15
Male	1.61 (0.69–3.74)	0.27	Male	1.40 (0.58–3.42)	0.46
ALT	1.01 (0.99–1.02)	0.45	ALT	1.02 (0.99–1.05)	0.19
HOMA-IR	1.14 (1.07–1.21)	< 0.001	HOMA-IR	1.03 (1.00–1.06)	0.03

*OR* odds ratio, *CI* confidence interval.

### Impact of insulin resistance on fibrosis in lean vs non-lean groups

**Fig. 4:**
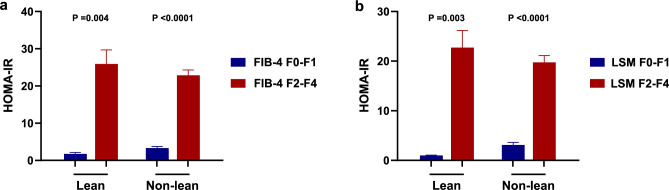
Association between the HOMA-IR and severe fibrosis (F ≥ 2) evaluated by FIB-4 index and liver stiffness measurement (LSM), stratified by the lean vs non-lean MAFLD.

As measures of insulin resistance seemed to be associated with the severity of liver disease, we next looked for correlations with an elevated HOMA-IR in lean and non-lean groups. A considerable proportion of patients with MAFLD are non-obese15. In this study, the positive relationship found between fibrosis severity (kPa and FIB-4) and insulin resistance was maintained in both lean and non-lean patients with MAFLD (Fig. 4). In multiple logistic regression analysis, HOMA-IR was clearly associated with significant fibrosis in lean (OR 1.06, 95% CI 1.01–1.12, p 0.03) and non-lean (OR: 1.04, 95% CI 1.02–1.07, p < 0.001) patients (Table 5).

**Table 5:** Multivariate analysis of factors independently associated with the presence of significant fibrosis measured by FibroScan stratified by lean vs non-lean.

Lean			Non-lean		
Variables	OR (95% CI)	p-value	Variables	OR (95% CI)	p-value
Age	1.00 (0.92–1.09)	0.96	Age	1.03 (0.99–1.08)	0.002
Male	3.11 (0.49–19.93)	0.22	Male	1.50 (0.80–2.83)	0.21
ALT	1.04 (0.99–1.10)	0.15	ALT	1.01 (0.99–1.02)	0.34
HOMA-IR	1.06 (1.01–1.12)	0.03	HOMA-IR	1.04 (1.02–1.07)	< 0.001

*OR* odds ratio, *CI* confidence interval.

### Insulin resistance

As measures of insulin resistance seemed to be associated with the severity of liver disease, we next looked for correlations with an elevated HOMA-IR. As expected, the HOMA-IR score correlated with age, diabetes, fibrosis stage (kPa and FIB-4), and elevated liver enzymes (p < 0.0001), but BMI was not associated with HOMA-IR (Table 6).

**Table 6:** Factors associated with elevated HOMA-IR.

Characteristics	HOMA-IR < 2.5	HOMA-IR ≥ 2.5	Univariate analysis
	(n = 159)	(n = 205)	p-value
Age (year) mean ± SD	44.04 ± 11.38	51.34 ± 11.30	< 0.001
Male N (%)	52 (32.7%)	87 (42.4%)	0.07
Diabetes N (%)	12 (7.5%)	106 (51.7%)	< 0.001
BMI (kg/m^2^) mean ± SD	31.43 ± 5.66	31.13 ± 5.29	0.63
Waist circumference (cm) mean ± SD	103.05 ± 10.42	105.64 ± 11.38	0.14
ALT (U/L) mean ± SD	33.44 ± 21.86	37.13 ± 20.18	0.01
AST (U/L) mean ± SD	29.99 ± 15.49	61.33 ± 27.46	< 0.001
Platelet (1000 cells/uL) mean ± SD	272.98 ± 64.95	210.07 ± 61.83	< 0.001
HOMA-IR mean ± SD	1.17 ± 0.59	18.53 ± 16.15	< 0.001
Hemoglobin A1c (%) mean ± SD	5.05 ± 2.68	7.43 ± 1.86	< 0.001
Total cholesterol (mg/dL) mean ± SD	192.20 ± 41.88	190.69 ± 36.59	0.94
LDL-cholesterol (mg/dL) mean ± SD	120.47 ± 37.51	146.72 ± 200.33	0.79
HDL-cholesterol (mg/dL) mean ± SD	46.40 ± 12.80	42.61 ± 8.55	0.42
Triglyceride (mg/dL) mean ± SD	143.09 ± 53.58	154.35 ± 77.13	0.80
Albumin (g/dL) Mean ± SD	4.20 ± 0.48	4.18 ± 0.47	0.86
Total bilirubin (mg/dL) mean ± SD	0.60 ± 0.34	0.94 ± 0.57	0.07
INR mean ± SD	1.00 ± 0.09	1.03 ± 0.07	0.26
High-sensitive C-reactive protein (mg/L) Mean ± SD	27.85 ± 64.98	4.75 ± 4.56	0.19
Lymphocyte (%) mean ± SD	7.33 ± 1.88	6.76 ± 2.53	0.32
ALP (IU/L) mean ± SD	80.63 ± 33.88	74.84 ± 32.92	0.59
FIB-4 mean ± SD	0.94 ± 0.54	4.47 ± 23.31	< 0.001
Significant fibrosis (LSM)	13 (9.4%)	157 (84.9%)	< 0.001
Significant fibrosis (FIB4)	3 (1.9%)	137 (66.8%)	< 0.001

## Discussion

In this study, our objective to assess the influence the effect of insulin resistance on patients with fatty liver disease. We reveal that for every unit increase of insulin resistance measured by HOMA-IR, there is a roughly 16% higher likelihood of experiencing substantial fibrosis, as determined by non-invasive scoring tests. This association remained significant regardless of the presence of diabetes or BMI status. Consequently, our findings support the value of insulin resistance is of added value in predicting fibrosis stage and implies the validity of integrating it into the diagnostic criteria for MAFLD.

Patients with MAFLD linked with metabolic dysfunction exhibit significant variability in the severity of fibrosis. Our investigation of a cohort of patients with MAFLD revealed that insulin resistance is directly linked to the extent of fibrosis, regardless of other factors. This discovery aligns with the findings of a previous study, including 361 Japanese non-diabetic patients with biopsy-confirmed MAFLD. The study demonstrated that HOMA-IR independently predicted the presence of advanced liver fibrosis^17^. Another longitudinal study, involving 32,606 participants and an 8-year follow-up period, found that the level of baseline insulin resistance, as measured by HOMA-IR values, was linked to the likelihood of fibrosis advancement in non-diabetic patients with MAFLD^18^. Metabolic score for IR, including HOMA-IR, was found to be associated with the incident advanced liver fibrosis^19^. Similarly, children with MAFLD who have higher HOMA-IR are more likely to have liver fibrosis^20^.

We found high HOMA-IR levels are considered a risk factor for significant fibrosis among MAFLD subgroups. A recent study of 1,727 adults from the National Health and Nutrition Examination Surveys showed that HOMA-IR can be used for assessing metabolic risks, early screening and monitoring disease progression in patients with MAFLD^21^ Similarly, a study of 1020 individuals demonstrated that a sequential algorithm incorporating AST and HOMA-IR levels improves fibrosis risk stratification among non-diabetic overweight/obese MAFLD individuals^22^. In a study of 2550 participants, those with advanced fibrosis were more likely to have higher HOMA-IR and advanced fibrosis was positively associated with subclinical atherosclerosis among MAFLD patients^23^. Interestingly, a recent study found that differences in subcutaneous adipose tissue transcript profiles in patients with vs without ≥ F2 fibrosis were explained by differences in HOMA-IR^24^.

The pathophysiology of hepatic fibrosis is significantly influenced by metabolic syndrome^25^. Metabolic syndrome-induced systemic inflammation worsens hepatic damage and speeds up extracellular matrix deposition, which eventually results in the onset and advancement of hepato-fibrosis^26,27^. The interplay between insulin resistance and lipid metabolism alteration influences MAFLD and MASH, which is a key precursor of fibrosis^27^. Our findings suggest that these pathological alterations begin to occur in the early stages of fibrosis before cirrhosis develops. Insulin resistance may contribute to the development of liver inflammation, which in turn stimulates the formation of fibrous tissue. Insulin resistance is an important component that can be modified and targeted with drugs to combat fibrosis in MAFLD^28^. The hyperinsulinemic clamp has traditionally been regarded as the most reliable technique for evaluating insulin resistance (IR), despite its drawbacks of being time-consuming, expensive, and challenging to use in clinical environments. The HOMA model was employed in this study as a proxy assessment of insulin resistance due to its simplicity, practicality, and affordability. These data indicate that it is important to examine insulin resistance, as measured by HOMA-IR, in individuals with MAFLD and include it in the diagnostic criteria. Notably, recent data suggest the superior utility of the MAFLD definition in identifying hepatic and extra-hepatic outcomes in adults and children^29–33^.

A significant fraction of patients with MAFLD are not obese, and this subgroup of patients appears to have distinct clinical and prognosis characteristics compared to their obese counterparts^32–35^. Our findings indicate that elevated insulin levels have a deleterious effect on the severity of fibrosis in both obese and non-obese patients with MAFLD. Furthermore, there was a significant correlation between HOMA-IR and the severity of fibrosis in both patients with diabetes and those without diabetes who have MAFLD.

Some limitations in our study need to be taken into account. First, we only studied Egyptian individuals. Consequently, our findings may not apply to other nations or ethnicities that exhibit distinct lifestyle trends. Fibrosis is assessed by transient elastography, which relies on inter-observer evaluation and experience, overcome by experienced technicians. All patients were recruited from tertiary hospitals, so there might be a referral bias. The utilization of a cross-sectional design hinders the ability to establish causal relationships. Furthermore, the data on lipid profile and waist circumference were not available for all cases in the cohort, so we could not compare the performance of HOMA-IR to other insulin resistance-related scores in predicting fibrosis. Finally, while our study group had sufficient statistical power to identify the primary outcome, its size was insufficient to conduct statistical tests for interaction between the different risk variables that influence the severity of liver disease.

In conclusion, insulin resistance is a reliable predictor of the severity of MAFLD, regardless of other factors. However, our research brings up the issue of whether excluding the measurement of HOMA-IR as one of the criteria in defining metabolic dysregulation in the definition of MASLD, as originally specified by the MAFLD definition, could decrease emphasis on addressing insulin resistance. Our findings suggest that metabolic factors, including insulin resistance, should be included in the clinical evaluation and diagnostic criteria for MAFLD.

## Data Availability

The datasets generated and analysed during the current study are not publicly available due to ethics considerations but are available from the corresponding author on reasonable request.
